# Biological trade and markets

**DOI:** 10.1098/rstb.2015.0101

**Published:** 2016-02-05

**Authors:** Peter Hammerstein, Ronald Noë

**Affiliations:** 1 Institute for Theoretical Biology, Humboldt-Universität zu Berlin, Berlin 10115, Germany; 2 Faculté Psychologie, Université de Strasbourg, Strasbourg 67000, France

**Keywords:** cooperation, mutualism, biological markets, partner choice, comparative advantage, principal–agent problem

## Abstract

Cooperation between organisms can often be understood, like trade between merchants, as a mutually beneficial exchange of services, resources or other ‘commodities’. Mutual benefits alone, however, are not sufficient to explain the evolution of trade-based cooperation. First, organisms may reject a particular trade if another partner offers a better deal. Second, while human trade often entails binding contracts, non-human trade requires unwritten ‘terms of contract’ that ‘self-stabilize’ trade and prevent cheating even if all traders strive to maximize fitness. Whenever trading partners can be chosen, market-like situations arise in nature that biologists studying cooperation need to account for. The mere possibility of exerting partner choice stabilizes many forms of otherwise cheatable trade, induces competition, facilitates the evolution of specialization and often leads to intricate forms of cooperation. We discuss selected examples to illustrate these general points and review basic conceptual approaches that are important in the theory of biological trade and markets. Comparing these approaches with theory in economics, it turns out that conventional models—often called ‘Walrasian’ markets—are of limited relevance to biology. In contrast, early approaches to trade and markets, as found in the works of Ricardo and Cournot, contain elements of thought that have inspired useful models in biology. For example, the concept of comparative advantage has biological applications in trade, signalling and ecological competition. We also see convergence between post-Walrasian economics and biological markets. For example, both economists and biologists are studying ‘principal–agent’ problems with principals offering jobs to agents without being sure that the agents will do a proper job. Finally, we show that mating markets have many peculiarities not shared with conventional economic markets. Ideas from economics are useful for biologists studying cooperation but need to be taken with caution.

## 1. Introduction

Cooperation among non-human organisms and human trade have in common that both result in net gain to all participants, so considering the two as different manifestations of the same basic phenomenon seems very promising. Going from trade to markets is then only a small step. The great advantage of adopting the trade and markets perspective on cooperation between individual organisms, of any kind, is that it provides inroads to a large body of knowledge already built up by the experts in trade and markets: the economists. The market paradigm was introduced into the study of cooperation among unrelated organisms in the early 1990s [1–5]. In hindsight, it is puzzling why the market idea, or at least its pivotal mechanism ‘partner choice’, was not part and parcel of the study of the evolution of cooperation from the start. This is probably no more than a historical coincidence: the question of the explanation of altruism dominated the scene from the 1960s onwards [6–8].

Prevention of ‘cheating’ or being ‘exploited’ by a partner during ongoing interactions was recognized as the main stumbling block on the way towards stable cooperative relationships. The cheating problem was first analysed with the help of what we hereafter propose to call ‘partner control models without outside options’ (see also [9]). In these models, two or more individuals are forced to interact and cooperation can be stabilized through partner monitoring and appropriate behavioural responses. Most of these models were based on variations of the two-player iterated prisoners' dilemma (IPD) and related games. Partner choice, switching and market monitoring are not considered. The idea of using the IPD comes from W. D. Hamilton (personal communication in [8]), but gained traction with a famous publication by Axelrod & Hamilton [10]. Biologists found the logic of repeated games so compelling that they apparently forgot to check every once in a while whether the empiricists had collected sufficient data to support it. A few rays of hope indeed emerged in the literature (coalitions among male baboons: [11]; blood meal sharing in vampire bats: [12]; grooming for support in vervet monkeys: [13]; egg-trading in hermaphrodite fish: [14]), but sooner or later doubt was cast on the validity of these and other examples (baboon coalitions: [15,16]; vampire bats: [17]; vervets: [16]; hermaphrodite fish: [18]). Given that almost half a century of research took place after Trivers's seminal publication, we consider the scarcity of convincing examples for the evolution of cooperation based on repeated games to be a sign of limited significance [e.g. 2,19–30], even though some more promising examples have recently emerged [31–34].

Until the early 1990s, few biologists scrutinized the way in which cooperating partnerships were formed, except in the context of mate choice and sexual selection. The term ‘mating market’ has indeed often been used, albeit mostly in a metaphorical sense. The metaphor is more than justified, because nobody in his right mind would lock a peacock and a peahen in a pen in an attempt to understand why the males have such long ‘trains’ and the females do not. This can only be understood if one sees their interaction against the backdrop of the peacock mating market, which is a lek system with intensive competition by males showing off their trains on a display arena to very fussy females. What is self-evident for reproductive cooperation should be self-evident for almost any form of cooperation where partners can be chosen: competition among agents offering the same good or service and choice by the other party among these agents are crucial to the understanding of how cooperative partnerships are formed.

In sharp contrast to the weak empirical support for cooperation merely based on the logic of repeated games, support for models including partner choice and partner switching, notably biological market theory (BMT), has been growing at a steady pace in the study of organisms as different as humans, non-human primates, birds and fish, and of numerous forms of interspecific mutualisms, a few examples of which we will discuss below. The burst of empirical examples shows that people started to look differently at cooperation through the lens of the BM paradigm, both in fields with a strong tradition of testing evolutionary models of cooperation (e.g. evolutionary psychology; behavioural ecology) and fields in which such models historically play a less important role, e.g. in the study of mutualisms, such as mycorrhizal symbiosis or the exchange of food against protection between plants and ants. In the two decades since we published our two ‘biological markets’ papers [3,4], various empirical and theoretical studies have been conducted on mutualism [35–41]. Some of these studies were primarily inspired by Bull & Rice [42] rather than our own and concentrated on ‘sanctions’, a specific form of partner choice in which large individuals (e.g. legumes) eliminate unproductive trading partners (e.g. rhizobia) [43–46]. Bull and Rice had proposed a fruit abortion mechanism by which figs eliminate fruit in which the fig wasp has claimed too many of the plant's ovaries for its own progeny. This abortion mechanism has since been described for numerous other obligate ‘nursery’ pollination mutualisms [47–53].

Some researchers interested in human cooperation and ‘fairness’ concentrated on the competition over the privilege to be chosen, rather than the choice mechanisms. Individuals are competing to obtain the reputation of not only a ‘good Samaritan’, but the ‘best Samaritan’ on the block in order to be preferred as cooperation partners, a strategy labelled ‘competitive altruism’ by Roberts [54]. In spite of its more general potential, this idea was almost exclusively picked up by people working on human cooperation and morality in general (e.g. [55–72]). In this short review, we cannot give proper attention to all of these human cooperation studies and will concentrate instead on the parallels and differences between human economics and recent developments in the biology of non-human cooperation.

We may give the impression here that partner choice models such as BMT are meant to replace partner control models. Partner choice is not an alternative to partner control, however. Partner switching means abandoning the current partner and hence has a similar controlling effect, from the viewpoint of the deserted partner, as ‘defecting’ in an IPD or related game. BMT is an extension rather than an alternative, but one that explains a number of aspects not considered in the original partner control models without outside options. Following a more model patching than conceptually extending approach, researchers have also added partner choice in various forms to more traditional partner control models (e.g. [73–77]).

## 2. What is a trade and where can it be found in nature?

The term trade refers to an exchange of goods and services for other goods and services, and the exchanged items are often referred to as ‘commodities’. Looking at the human world, the existence of money appears to greatly facilitate most forms of trade, but although grooming in primates may sometimes serve functions similar to Aristotle's ‘unit of account’ [78], human monetary systems are not paralleled in nature. In contrast, the following observation about human trade is of great importance to biology. Trade results mainly from specialization and division of labour. Most people specialize on a small aspect of production, trading for other products. This gives biologists a hint where to expect trade-like phenomena in the world of animals, plants and other organisms. Strong specialization and division of labour are most likely to be found in mutualistic interactions among separate species. The reason is that species typically differ in their resource requirements and relative ability to provide goods or services. These differences can serve as pre-adaptations promoting the initial evolution of biological trade, and they can reduce competition between potential trading partners. Last but not least, the genetic separation between species facilitates strongly the evolution of further specialization (or even its initiation) among interspecific trading partners. These ideas are not unparalleled in economics. In the early days of economics, Ricardo [79] developed a theory of international trade which suggests that nations should invest their resources mainly in industries where they have a comparative advantage in competition with other countries, and trade with the latter to obtain the products they stopped producing.

Even within a given species, there are genetic or situation-specific differences between individuals on which trade can be built. The trade can, for example, take place between a female and a male, between males of different age and status, or simply between an individual in need of ‘scratching its back’ and a partner that can actually reach that back.

### (a) Well-studied examples of trade between species

E1. Trading nitrogen for help with respiration—the symbiotic relationship between rhizobia (nitrogen-fixing soil bacteria) and their plant hosts (legumes). Located in root nodules, these bacteria make atmospheric nitrogen available to the host and receive in return carbohydrates, proteins and oxygen for their respiratory metabolism [45].
E1. Trading minerals for carbohydrates—mutualistic interaction of fungi with the roots of vascular plants in mycorrhizal symbiosis. While receiving carbohydrates from the roots, the fungi provide minerals in return. They convert demineralized phosphorus and make it available for uptake by their plant hosts [80,81].
E3. Trading ‘board and lodging’ for defence—the animal–plant symbiosis between bullhorn acacias and some ant species of the genus *Pseudomyrmex*. Bullhorn acacias offer shelter and nutrition to the ants, feeding them with extrafloral nectar and offering them large hollow thorns for nesting. The ants reward this effort by defending these trees against a variety of herbivores [82,83].
E4. Trading pollination for food—obligate pollination mutualism between yucca plants and certain yucca moths. The adult moth pollinates yucca flowers but also lays eggs inside the ovaries. Her larvae feed on the plant's seeds [51].
E5. Trading light for nutrition—bioluminescence generated by luminous bacteria (*Vibrio fischeri*) in the light-emitting organ of the Hawaiian bobtail squid *Euprymna scolopes*. The bacteria associate with juvenile squids and are nourished by their host. They enable the squid, in return, to emit light from its ventral surface. This serves the squid to hide its silhouette when viewed from below—a camouflage strategy known as counterillumination [84–86]
E6. Trading cleaning services for food—the mutualism between cleaner fish and a variety of fish species. On coral reefs, wrasses of the genus *Labroides* remove dead skin and ectoparasites. They receive nutrition this way, but also take more than a ‘gentle bite’ from the surface of their ‘clients’ occasionally [9].
E7. Offering repair after causing damage—destruction of a host's tongue followed by functional replacement of the destroyed organ. The aquatic isopod *Cymothoa exigua* enters fish and causes tongue atrophy through parasitic activity. It then attaches itself to the remaining stub and serves as a living ‘tongue prosthesis’, thereby raising the host's fitness prospects given the damage has been done already [87]. In this way the parasite actually supports its host and reaps nutritional benefits in return—an extreme case of a forced trade.

### (b) Examples of trade between members of the same species

E8. Offering parts of a territory for support with reproduction—cooperation between males of different status groups. In the songbird lazuli bunting (*Passerina amoena*), territorial males give immature-looking males (i.e. males with delayed plumage maturation) access to peripheral parts of their mating territories and are compensated through reproductive benefits that result from copulations with the subtenant's mate [88].
E9. Trading food for sex and paternity—in the spider *Pisaura mirabilis* males offer silk-wrapped prey to the female during courtship. This effort improves their chances of being accepted as a mate, accelerates female oviposition and increases paternity [89].
E10. Trading grooming for agonistic support among adult male chimpanzees—in groups with steep hierarchies, high-ranking males trade their support to lower-ranking males against grooming by those males [90].
E11. Trading grooming for acceptance as a mate—in long-tailed macaques, males groom females before mounting them and groom longer when there are more adult males in the vicinity [91].

## 3. The theory of biological trade, and how it differs from economics

In order to understand the evolved nature of a biological trade, the evolutionary stability of the traders' strategies needs to be examined [3,92]. It is perhaps trivial, but nevertheless important to realize in this context that cooperation as such is an end result of an interaction and hence itself not under selection. Under selection are mechanisms at the individual level that may or may not result in cooperation. In a first step of the analysis, biologically relevant sets of strategies have to be identified that capture the traders' behavioural options. This—admittedly difficult—step includes answering the following questions: how can power be exerted and what can a trader control? What commodity can the trader give (or lose to) a partner in what quantity under different circumstances? How can traders sanction (or dispose of) those partners who fail to meet their needs? How can partners be searched, assessed and recruited? In a second step, the fitness consequences of strategies need to be studied in detail. How much does it cost, for example, to sanction a trading partner, and how strong is the effect of sanctioning?

### (a) What are the terms of contract?

The concept of evolutionary stability strongly shapes our views on natural transactions. Most importantly, it is well known from evolutionary game theory that many potential forms of trade and cooperation can hardly evolve because they include no safeguards against cheating and exploitation. Even in cases where cooperative trade actually has a chance to evolve, there is often a potential for higher net benefits that cannot be fully exploited. The reason for this is that evolutionarily stable trades can typically be characterized as Nash equilibria, and the latter almost generically fail to be Pareto efficient (a given trade is called Pareto efficient if none of the traders can improve their pay-off without worsening that of a partner; all other trades involve an unrealized potential for cooperation).

This lack-of-efficiency problem that is reflected in the original game theory developed by mathematicians and economists is often more or less explicitly defined away. Cooperative game theory (a field of little relevance to biology because it assumes cooperation instead of explaining it) simply assumes the existence of Pareto efficiency as part of its methodological foundation [93]. In a similar way, conventional economics typically considers models of trade and markets in which the goods and services are subject to complete contracts that are enforceable at no cost to the exchanging parties [94]. This means that the explicit terms of the exchange cover all aspects of the trade that are relevant to the trader, and, once decided upon, these terms are not subject to cheating. Of course, by making such strong assumptions, the way is paved for Pareto efficiency. In a Prisoner's Dilemma-like situation, for example, the traders would sign a contract in which both commit themselves to choosing the cooperative strategy.

Human contracts are unique in nature because no other species has developed institutions as powerful as our laws and their enforcement, through which almost any trade could, in principle, be protected against breach of contract. In the world of non-human organisms, however, there are no policemen to arrest traders who play against the rules and no judges to sanction them. Most biological trade can thus be expected to have self-stabilizing properties in the sense that the actions of all traders are compatible with their individual ‘fitness incentives’ [95]. These self-stabilizing properties determine what might be called the ‘biological terms of contract’. The scope for biological trade thus seems very small in comparison with human trade—most human contracts would not be self-stabilizing in nature. Conventional theory from economics, therefore, does not apply all too well to biological trade. However, modern economists have learned that human traders are not as committed to their contracts either. This puts modern economics in much closer proximity to the life sciences [94].

### (b) How is biological trade self-stabilized?

Bobtail squids (example E5) provide a particularly intriguing example of self-stabilized trade. They have to ensure that the commodities they offer are selectively transferred to cooperative partners, that is, to bacteria from which they receive light in return [84,96,97]. This is difficult to achieve, however, since a great variety of bacteria exists in the sea and, in addition, the association between the squid and its partners is formed anew in each generation. Shortly after hatching, the squid recruits its first bacterial ‘passengers’. A population of *Vibrio fisheri* then quickly builds up in the light organ. After this initial recruitment, no more bacteria are ‘admitted’ and every day 90–95% of the bacterial population are pushed out by the squid [84]. But what enables the squid to select the luminescent bacteria, *V. fisheri*, and why should these actually produce light?

While recruiting bacteria as partners, the squid uses antibacterial defences to protect its entry sites. This apparently paradoxical way of opening the door for bacterial guests makes it possible to receive just a few of those that are ‘welcome’ [96]. The wanted guests, *V. fisheri*, seem better able than unwanted bacteria to resist the squid's defences. Furthermore, by letting just a few bacteria in, the squid probably makes it harder for unwanted invaders to hide among *V. fisheri*. Once in the light organ, the selected bacteria produce the enzyme luciferase that catalyses an oxidation reaction through which blue-green light is generated. The squid senses this cooperative behaviour and changes some of its gene expression patterns according to the perceived luminescence [98]. By mechanisms that are not fully understood yet, the squid manages to eliminate dark individuals of *V. fisheri* from its light organ [96]. This creates the incentive for *V. fisheri* to actually produce light and stabilizes the trade. The evolution of this now highly advanced mutualism was probably facilitated by the fact that luciferase interacts with host defences when it generates luminescence by indirectly disrupting the host's production of reactive oxygen species [84,97]. Hence, the bacteria's answer to the host's antibacterial defences almost inevitably generates the light that benefits the host.

Returning now to the general question of how trades are self-stabilized, the evolution of increased dependence of trading partners on each other can strongly protect trade from cheating [95]. This is nicely demonstrated by the example (E3) of the mutualism between acacia trees and their ant defenders. The extrafloral nectar offered to the ants contains glucose and fructose but virtually no sucrose [99,100]. This makes the nectar unattractive to unspecialized ant species because diet choice of ants in general seems to be strongly driven by sucrose content. The mutualistic *Pseudomyrmex* ant species, however, depend on the tree's extrafloral nectar, having lost the ability to digest sucrose [100]. The ‘ant bait’ is offered away from the tree's flowers, because preventing pollinator visits to flowers is certainly not in the interest of the tree. It is a likely evolutionary scenario that the acacia trees altered their sugar production, because this had the advantage of making them less attractive to non-mutualistic ants, and that this in turn created the need for the mutualistic ants to specialize. Finally, being well defended by an ‘ant army’, the acacia seems to have been under selection to restrict its ‘budget for self-made defences’. This budget cut manifests itself in the tree's leaves, which do not contain any of the alkaloids used for defence by other Acacia species. What results from these evolutionary steps is a mutual dependency between ant and acacia that is strong enough to create a high degree of ‘common interest’ [92]. Common interest is, of course, the best prerequisite for a trade unhampered by cheating and exploitation.

Many mutualistic relationships are not stabilized by common interest. In the yucca plant example (E4), the plant needs fertilization services of its mutualistic moth but a moth individual would, in principle, have fitness benefits from depositing an excessive amount of eggs, thereby overexploiting the plant. Yucca plants, however, actively limit this option for fitness gains. They abort those flowers [50,51] into which the moth has laid excessive amounts of eggs. This stabilizes the trade but it leaves one question open. What caused the evolution of selective abortion? Presumably, it did not evolve in the first place for reasons of sanctioning, since the first sanctioning yucca plants would not have benefited from the effects of their sanctions on moth evolution. It is much more likely that the plants originally had a more generally used shut-off mechanism that prevented the allocation of resources into damaged flowers and fruit. This mechanism would have had sanctioning effects as a by-product and could therefore be considered as pre-adaptation for the self-stabilizing trade between yucca plant and yucca moth. Similarly, in rhizobia–legume mutualisms (example E1), the plants tend to selectively stop the resource flow into parts of their roots, in which rhizobia draw on the plants' resources without providing nitrogen in return. This could be demonstrated for soya beans in experiments with rhizobia acting in an N_2_-free atmosphere [101]. The general question of how sanctions can evolve and how they work has received much attention in recent years [46,95,102–104].

## 4. The theory of biological markets

As discussed above, the bobtail squid example (E5) provides an interesting case of a trade, where one trader has evolved mechanisms to choose the best partners for a beneficial exchange. By putting up its defences, the squid implicitly exerts choice for bacteria prepared to ‘pay the cost’ of evading these defences [84,105]. Conversely, by paying their cost, the chosen bacteria generate light as a by-product, thus delivering their luminescence service to the squid. Owing to partner choice, this trade is self-stabilizing—no need for signing a contract as in conventional economics.

The importance of partner choice in the evolution of cooperation was recognized early [1,2,16,18,42,106], followed by first attempts to develop a theory of biological markets, where an exchange of materials and services takes place and the trading organisms (or at least the organisms belonging to one class of traders) have some choice with whom to trade [3,4]. To illustrate why a market approach is important, consider the following situation. When we buy a basket of cherries, the interaction with the farmer is mutually beneficial: we receive a commodity while the farmer gets a reward in return. Mutual benefits, however, do not imply that they are worth the trade. We tend to refuse a particular trade if we know that a better deal can be obtained elsewhere, or if we can pick the cherries ourselves with little effort.

BMT is needed more generally to reflect on the following issues that are often important in studies of trade-like cooperation:

— competition among biological traders can reduce the ‘price’ of a commodity (the price need not be the same for all individuals) and it can lead to specialization;
— supply and demand can influence the net benefits from a trade;
— traits can evolve solely because of market selection induced by partner choice in trade-like situations (an analogy with sexual selection);
— the market can limit the scope for false advertising; and
— trading partners can be controlled by the threat of partner switching.

In the subsequent sections (a–c), we discuss a number of fundamental achievements of BMT that all relate to several of the issues just listed.

### (a) Modelling mycorrhizal symbiosis in the tradition of Cournot

Guided by the empirical knowledge on mycorrhizal symbiosis (example E2), Wyatt *et al*. [41] developed a biological market model of broad importance. They investigated how natural selection induces competition between partners over the price at which they provide goods, and how this determines the ‘terms’ of mutualistic trade as well as the patterns of trade-specialization. Let us take a closer look at their model, which illustrates the validity of the biological market analogy particularly well.

There are two types of traders in the model: plants and fungi. Both are in principle able to acquire both phosphorus and carbon directly but it is not assumed that this double capability is maintained by natural selection. In fact, mycorrhizal fungi in current symbioses do not directly acquire carbon but the model allows the question ‘what if these fungi had been able to acquire carbon in the evolutionary past?’

In the model, both plants and mycorrhizal fungi require carbon and phosphorus for their own growth and both can directly acquire these goods up to maximal levels (for simplicity, the maximal levels are kept constant; in particular, one or two of these levels can be set to zero). The trade-off between the resources invested into carbon and phosphorus acquisition is linear, which means that if *x* per cent of the carbon maximum level are acquired, the acquisition of phosphorus is limited to 1 − *x* per cent of the phosphorus maximum level. Each plant has a finite number of fungi as trading partners and each fungus a finite number of plants. Plants transfer a certain proportion of their directly acquired carbon to the fungi they trade with and the fungi deliver a proportion of their directly acquired phosphorus to their plant partners. Individuals compete simply by simultaneously setting the quantities they supply. This assumption was also made by Cournot in his famous model of human markets with a small number of firms under competition [107]. In analysing his model, Cournot calculated what is now called a Nash equilibrium [108,109]. This happened more than a century before Nash introduced this concept, which incidentally is closely related to the biological notion of an evolutionarily stable strategy (ESS; [92,110]).

With a few more specifications, the biological market under consideration can be subjected to analysis for evolutionarily stable strategies. The most important questions are ‘will trade be maintained by selection, and what are the evolutionarily stable strategies employed during sharing between partners?’ Wyatt *et al*. [41] showed for their model that evolutionarily stable trade can exist in which individuals ‘produce goods’ and allocate them among partners of the other species in direct proportion to the relative amount of benefits they receive from each partner. This allocation principle is called ‘linear proportional discrimination’ and its justification relates, in particular, to the assumption that increasing the level of an individual's resources gives decreasing marginal fitness returns.

Once the considered community of traders has adopted linear proportional discrimination, all individuals receive the same exchange rate for the resources traded. Before Wyatt *et al*. conducted their study, it had been an open question whether natural selection could induce such invariant exchange rates in an evolutionarily trade. The result demonstrates impressively how close an analogy can be drawn between certain markets in biology and economics. At least in this model, natural selection can lead to all individuals receiving the same market exchange rate for their goods, as it happens on a human central market place.

How well is this interesting theoretical result supported by facts on mycorrhizal symbiosis? Several studies have shown that in mycorrhizal symbiosis, an increase in the quantities supplied leads to an increase in the quantities received [101–112], but is not known yet whether the symbionts follow the rule of linear proportional discrimination. Two more insights gained from the model relate in interesting ways to the facts [41]. First, the trade in mycorrhizal symbiosis can only be maintained under selection when individuals are exposed to a sufficiently large number of competitors within their own trader class. As the authors emphasize, there is empirical support for this result because individuals in mycorrhizal communities typically trade in large networks of partners [113,114]. Second, according to the model's analysis, mycorrhizal fungi would probably have lost the ability to directly acquire carbon in response to market forces (reminiscent of the loss of self-defence in the bullhorn acacia mentioned above). Natural selection would have caused this loss through between-fungi competition for the benefits obtainable from plant partners. The model thus offers a potential explanation for structural aspects of the mycorrhizal community known from empirical studies.

### (b) The ‘principal–agent problem’: how it can be resolved in nature and why it can impact trait evolution

In human economic affairs, an employer hires an employee in order to delegate activities that serve the ‘boss’. But even with employment contracts and thorough supervision, it is difficult to bind the hired person to do the actions that are optimal from the employer's point of view. This is a version of the ‘principal–agent problem’ known from recent developments in economics [115]. The ‘principal’ (employer) is in the position to give the job to the ‘agent’ (employee) but has limited power to subsequently control that agent.

A similar problem seems to occur in the animal world [94]. The lazuli bunting example (E8) may serve as an illustration. Here, the male owner of a mating territory acts like an employer who offers a job to younger males. Once accepted, the young males are allowed to establish a territory in the high-quality area directly adjacent to the territory of the boss and attract a female who then mates with the boss and his employee, so that both sire some of her offspring. Finally, the young male and his female raise their offspring as is usual in Passerines with bi-parental care. This is a trade with obvious fitness benefits to both the ‘principal’ and the ‘agent’—but why is it self-stabilizing? Since the young male cannot distinguish his own offspring from that of the territory holder, his efforts to raise the latter's offspring are simply a by-product of working hard for his own progeny. Conversely, if the boss attempted to completely monopolize reproduction, the employee would have good reasons to ‘quit the job’. Finally, the female, in need of help with parental care as well as access to a territory rich in resources, has a strong fitness benefit from mating with both the boss and the employee.

How does the boss prevent his employees from sneaking up to his own mistress to perform a little extra-pair copulation? He does this by rigid partner choice: the yearling males, which arrive at the breeding grounds later than the fully adult males, are rather varied in their plumage: some are almost as lazuli blue as the full adults, some are almost as dull brown as females (technically speaking, the latter show the highest degree of ‘delayed plumage maturation’). Established bosses only allow the dullest looking males—for which their own mistresses almost certainly turn up their noses—in their immediate vicinity. Hence, by partner choice exerted by the bosses, there is selection for delayed plumage maturation in the yearling males, a phenomenon essentially predicted by Noë & Hammerstein [3] and now called ‘market selection’, a term used for selection owing to partner choice in contexts other than mate choice [9].

### (c) Why the Walrasian market model is of little relevance to biology

The conventional market model in economics is termed Walrasian after Léon Walras (1834–1910), one of the founders of neoclassical economics [116]. This model assumes that enforceable contracts and can be used, for example, to derive the ‘law of supply and demand’. The law states that in a market economy, the forces of supply and demand push the price to a level at which the quantity supplied and the quantity demanded are equal, a result called market clearing. Given the biologically unrealistic assumption of enforceable contracts made in the theory of market clearing, biologists have good reasons to be critical of this law. Even in economics, the Walrasian model has for the most part been superseded. The new market theory is quite different from the old and takes as its foundational assumptions the incomplete nature of contracts (biologically speaking, the possibility of cheating, exploitation, etc.), as well as the traders' limited information about the trades being offered and accepted by other traders [94]. The new post-Walrasian microeconomics provides, for example, models of labour markets, credit markets and markets for goods of variable quality, in which market clearing does not occur [115]. Market clearing is also very unlikely in most biological markets, because the numbers of traders in each class are usually not determined by the cooperative interaction alone (with the possible exception of some obligate mutualisms and mating). It is unlikely that the total quantity of each commodity is held constant when the number of traders providing it changes (relatively) quickly. Drought, for example, may kill a plant species but not its pollinators. The latter are more likely to switch to another host than to starve.

### (d) The role of supply and demand in biological mating markets

Despite the inapplicability of the strict law of supply and demand, these two quantities often matter for the understanding of biological trade. The so-called mating market, where male and female partners offer each other commodities like egg and sperm, may serve as an example. Since there are no wedding contracts in the animal world, females often receive very little from males they mate with, even if competition is asymmetrical so that females can choose between a variety of ‘male offers’. From a neoclassical economics perspective, females should then perhaps be able to claim a ‘package deal’ from a male partner, including substantial commodities in addition to the sperm that is so readily available. For quite a number of species, female mating behaviour appears to be influenced by this logic. An imbalance of supply and demand can indeed have a strong impact on biological trades, but only as long as appropriate self-stabilizing contracts have a chance to evolve. This seems to be the case in the spider example (E9) where the female typically receives a prey as ‘nuptial gift’ from her male partner. Males offering prey have better mating success, longer copulations and longer palpal insertions than those without. The gift under consideration is neither an investment in the offspring, nor does it serve to avoid being eaten by the female. It mainly serves the purpose of gaining the female's acceptance [89].

Unlike this case of a self-stabilizing trade, mating occurs in many species without gift giving. Males of *Drosophila melanogaster* even add a toxic protein to the seminal fluid through which they reduce their mate's lifespan [117]. This protein modulates the female's reproductive physiology to the male's advantage, enabling him to sire more of her offspring at the expense of her lifetime reproductive success. In principle, the mating market could force male *Drosophila* to be less toxic to their partners but—in the absence of binding contracts—this would require a pre-mating test for toxicity of the seminal fluid.

Many of the questions economists might ask about biological mating markets have already been answered in biology. One question concerns the market entry problem: that is, on which side of the mating market—male or female—an organism should operate. This problem has, of course, been solved by sex ratio theory [118–120]. According to this well-known theory, males and females are often produced in very similar numbers. Taken together, Fisherian sex ratios and anisogamy (egg and sperm typically differ in the amount of resources needed for their production) are the main causes for the oversupply of sperm in animal populations. This oversupply facilitates the evolution of female choice and the resulting sexual selection on male traits. Sexual selection allows females to let some of their demands govern the evolution of heritable male traits—a positive feedback on the evolutionary time scale. Quite surprisingly, however, strong sexual selection can also feedback negatively on female success in ‘trading with males’ because it can ultimately weaken the female's strategic position in the evolutionary conflict over parental investment. The argument is as follows [121]. If the adult sex ratio is unbiased and females are choosy, some males will not meet the females' choice criteria so that more females than males become parent of at least one offspring. In this case, male parents will have on average more offspring than female parents. The reason is that (in diploid organisms) every offspring has exactly one parent of each sex. Male parents are then likely to pay a higher ‘opportunity cost’ than female parents if they engage in parental care, making males less likely than females to evolve parental care—an ultimate consequence of partner choice.

### (e) Monopolistic competition

A provider of commodities that cannot be substituted by other providers has a monopoly and can sell products way above the price one would observe on competitive markets. Cleaner fish (example E6), for example, can have a monopoly with respect to local clients who are not sufficiently mobile to exert choice between different ‘cleaning stations’. As shown by Bshary [122], such local clients get an inferior service compared to those who are able to switch partners. With respect to these more mobile clients, the cleaner fish do not hold a monopoly and seem forced by the market to offer a better deal. Fruteau *et al*. [123] demonstrated a similar effect in experiments with wild vervet monkeys, where they enabled low-ranking females to provide food to their groups, for which they received grooming benefits in return. These benefits were highest when a single female had the monopoly of food provisioning, but were significantly reduced after a duopoly of two food providers was created experimentally.

### (f) Comparative advantage: a key concept in the theories of trade, competition and signalling

One of the most useful concepts for biologists to learn from economics is that of ‘comparative advantage’, first described together with a numerical example by Ricardo [79] in his theory of international trade, and used for the first time in the context of biological markets by Schwartz & Hoeksema [39]. This concept is quite intricate but seems erroneously trivial to all those who do not fully understand it. Ricardo himself offered the following scenario to illustrate what the term ‘comparative advantage’ really means. He looked at two countries, Portugal and England, and assumed both Portugal and England could produce the goods wine and cloth. Ricardo considered the amount of labour as the ‘cost’ of producing either of these goods and treated the quantity of output produced per worker as the country's productivity. He assumed that, compared to England, Portugal was more productive in producing both wine and cloth. Naively, one would then think that with respect to these goods Portugal has a productivity advantage over England, and England thus could not succeed in profitably selling cloth to Portugal. But this tempting thought is based on comparing absolute productivity between countries. Ricardo's important contribution to economics was to show how misleading such a comparison of absolute productivity can be.

While, in his example, England is assumed to be an inferior producer of both goods, it could nevertheless turn out to be beneficial for both countries to specialize and engage in a ‘wine for cloth’ trade—of course, with Portugal specializing on wine. Why would this be so? Ricardo assumed that instead of having an absolute advantage, England had a comparative advantage over Portugal in cloth production. This means that England would lose less of its wine production than Portugal if it reallocated work force from wine to cloth production in order to generate one more unit of cloth—a very plausible assumption, compatible with Portugal's absolute advantage. Speaking more abstractly, a country has a comparative advantage relative to another in producing a good if it can produce this good at a lower opportunity cost.

In Ricardo's scenario, both countries have a comparative advantage over one of the goods and, as he demonstrated in his numerical example, both benefit from specializing production on the good for which they have the comparative (not necessarily absolute!) advantage. Effects of this kind have been demonstrated for a variety of biological models. For example, in the model of mycorrhizal trade (discussed above, [41]), plants and fungi are comparable to Ricardo's countries and have resources (Ricardo's work forces) they can invest in the acquisition of two different trading goods (carbon and phosphorus). The fungi could originally have resembled England and its wine production in that they might have had less of an opportunity cost than plants in producing phosphorus. This would have fostered specialization on phosphorus, as shown by Wyatt *et al*. [41].

We wish to emphasize at this point that the concept of comparative advantage helps biologists sharpen their notion of pre-adaptation. A naive view would have been that if one of two species evolved to become a specialist for the production of a particular good, it would likely be the one that is better able to produce that good in the first place. This view is incorrect from a theoretical point of view because the—intuitively more demanding—comparative advantage is usually the key to specialization. Interestingly, this argument is valid beyond the theory of biological markets. Riechert & Hammerstein [124] presented an evolutionary game involving two plant species with root competition. In their ecological model, both species extract resources from two strata of the soil if they exist in isolation but coevolution leads to character displacement and one then becomes a specialist for the less rewarding stratum. Riechert and Hammerstein showed that the specialist in an evolutionarily stable equilibrium is the species that has a comparative advantage in using the poor resource.

Advertising is another field where the comparative advantage provides theoretical insights. Suppose that one organism sends a costly signal to ‘show off’ an otherwise invisible quality. Two such signallers may then send signals of different intensity. If they play an ESS, the receiver can safely infer the following on how their qualities differ. The player whose signal is stronger by an amount *D* must have a comparative advantage in producing this additional amount *D*. It takes only a few lines of algebra to demonstrate that the comparative advantage in question is a necessary condition for evolutionary stability in the signalling game [94]. This approach provides the easiest access to the so-called handicap principle, first suggested to biologists by Zahavi [125] and later made precise by Grafen [126]. Bowles & Hammerstein [94] describe a close relationship between this principle and Spence's theory of signalling in economics [127]. The biological handicap principle has been refined by taking life-history aspects into account. Condition-dependent indicators of quality may, for example, become more reliable with age, so that older males can reveal more information in their sexual displays and females may evolve a preference for older males [128].

## 5. Outlook

### (a) The time frame of book-keeping

In order to choose the most profitable partner(s) from a larger set of potential partners, an agent has to be able to compare them. There are many mechanisms by which this can be done, depending on the species involved. A straightforward way of comparing and choosing is to trade with a large number of partners simultaneously and then cut the ties with those that yield the lowest profit. This kind of mechanism can notably be found in mutualisms in which a single, large agent interacts with a large number of small members of another trading class, e.g. plants with rhizobia or bobtail squids with bioluminescent bacteria. ‘Sanctioning’ [43] is the most drastic form of selective partner elimination as it normally results in the death of the smaller partners. A much more hazardous way of choosing partners is by relying on advertisements that signal the potential partners' quality and/or intentions without any immediate benefit for the choosing party. Here, the problem of dishonest advertising looms large [129], but see above for a solution. In between those extremes are many ways of discriminating among partners with whom the choosing agent has a series of interactions or a single, but long-lasting interaction of the symbiotic type. In such cases, multiple partners can often be evaluated and compared over longer time frames. The length of those time frames has been a source of confusion, however, notably when cognitive mechanisms are involved in the evaluation process, e.g. in primates and other vertebrates. The pivotal question here is: which mechanisms do the agents use for their ‘book-keeping’? de Waal [130] distinguished (for non-human primates) between ‘symmetry-based reciprocity’, ‘calculated reciprocity’ and ‘attitudinal-based reciprocity’. Relevant to the present question are the latter two.

Calculated reciprocity assumes a relatively precise accounting on investments done and benefits received. It requires considerable cognitive capacities including a good memory and has, unsurprisingly, received very little empirical support. ‘Attitudinal-based’ reciprocity, which has also been called ‘emotional book-keeping’ [131], assumes that a current account is kept by means of mechanisms such as neuro-hormone titres that are adjusted a little bit during each positive or negative interaction with the partner. The time frame over which this process takes place is long rather than short and a single run-of-the-mill interaction is unlikely to have a great effect on the agents' ‘attitude’, in sharp contrast to agents playing a tit-for-tat strategy. The same mechanism can also play a role in a partner choice context, where it has been called ‘attitudinal partner choice’ [123]. What this means is that partner choice can also be effectuated over time frames that are (very) long relative to the species lifespan.

A widespread misconception, notably in primatological circles, is that BMT is only about tracking changes in supply and demand ratios over very short time frames. This is probably owing to a confusion between the phenomenon itself and the methods used to show its existence. In order to validate BMT, it is often important to show, by observation but better still by experiment, that animals react to changes in supply and demand. Life is short and PhD-studies even shorter, so researchers do this by using strong changes in supply and demand that are likely to elicit immediate strategic adaptations (e.g. [123,132]). Under normal circumstances, however, the attitudes of primates towards their group-members do not change overnight and may even be stable over lifetimes. Primatologists love the term ‘social bond’ in this context, often suggesting that this signifies an alternative to interacting according to market forces, but they should not forget that forming bonds between unrelated members of a primate group initially results from partner choice too, unless one assumes partnership formation by external forces or chance events, and that partner switching among close friends is not unheard of [16]. BMT simply suggests that mechanisms of partner choice are adapted to the time frames over which the supply and demand ratios of the relevant commodities tend to fluctuate.

### (b) Use of force is excluded from theoretical models, but a common phenomenon on all markets, biological or not

Primate mating markets are good examples of markets on which traders can obtain a commodity either by providing a service (usually grooming) or by the use of force [133,134]. In the early market papers, we wrote that commodities traded on biological markets ‘could not be taken by force’ [1] and that ‘individuals do not compete over access to partners in an agonistic manner’ [3]. By contrast, in sexual selection theory—the usual framework in which mating markets are placed—intra-sexual competition is considered to be one of the two major drivers of selection besides mate choice. We concentrated on partner choice and excluded the use of force in order to make market models more easily traceable. That does not mean, however, that the use of force is completely incompatible with the market metaphor. Theft and robbery are inextricably bound up with many markets and the primarily stolen commodities are those that got their high value owing to market forces. Theft is also rather common in human societies; around 11% of US citizens are estimated to commit some shoplifting during their lifetime [135]. Nevertheless, mainstream economics does not deal with it in its models and we followed that tradition. It would be good, however, if this set of strategies were taken seriously too in market models. For BMT, this would notably mean that parasitic strategies get their proper place in the strategy space.

### (c) Partner choice in team formation

BMT in its present form focuses on dyadic trade, i.e. the actual exchanges of commodities are between two individuals, usually belonging to two different trader classes. The core mechanism that distinguishes BMT from simple partner control models is partner choice. A similar distinction can be made for cooperation in which teams consisting of more than two agents have to act in a coordinated fashion in order to make cooperation successful. The dynamics of team formation and maintenance is far from trivial, partly because partner choice as a mechanism of team formation can take many more forms. Take, for example, the addition of one member to a team: is this done by unanimous consent among the existing members, by majority rule, or by a single ‘leader’? The same can be said about the exclusion of members. Moreover, it is not only single members that can switch teams and thus play out teams against each other, but also sub-sets of teams, alliances that form within teams. Most of this plays an obvious role in human societies—one only has to look at politics and government formation, for example. Non-human agents, from bacteria to whales, also form teams for all kinds of purposes [136], but hitherto more attention has been paid to the mechanisms of partner control in existing teams than to the mechanisms that play a role in the formation and changes of composition of non-human teams. We hypothesize that partner choice is a pivotal mechanism in this domain too.

## References

[1] Noë R, van Schaik CP, van Hooff JARAM 1991 The market effect: an explanation for pay-off asymmetries among collaborating animals. Ethology 87, 97–118. (10.1111/j.1439-0310.1991.tb01192.x)

[2] Noë R 1992 Alliance formation among male baboons: shopping for profitable partners. In Coalitions and alliances in humans and other animals (eds Harcourt AH, FWaal BMD), pp. 285–321. Oxford, UK: Oxford University Press.

[3] Noë R, Hammerstein P 1994 Biological markets: supply and demand determine the effect of partner choice in cooperation, mutualism and mating. Behav. Ecol. Sociobiol. 35, 1–11. (10.1007/BF00167053)

[4] Noë R, Hammerstein P 1995 Biological markets. Trends Ecol. Evol. 10, 336–339. (10.1016/S0169-5347(00)89123-5) 21237061

[5] Peleg B, Shmida A, Ellner S 1992 Foraging graphs: constraint rules on matching between bees and flowers in a two-sided pollination market. J. Theor. Biol. 157, 191–201. (10.1016/S0022-5193(05)80620-4)

[6] Hamilton WD 1964 The genetical evolution of social behaviour I. J. Theor. Biol. 7, 1–16. (10.1016/0022-5193(64)90038-4) 5875341

[7] Williams GC 1966 Adaptation and natural selection. Princeton, NJ: Princeton University Press.

[8] Trivers RL 1971 The evolution of reciprocal altruism. Q. Rev. Biol. 46, 35–57. (10.1086/406755)

[9] Bshary R, Noë R 2003 Biological markets: the ubiquitous influence of partner choice on the dynamics of cleaner fish–client reef fish interactions. In Genetic and cultural evolution of cooperation (ed. Hammerstein P), pp. 167–184. Cambridge, MA: MIT Press.

[10] Axelrod R, Hamilton WD 1981 The evolution of cooperation. Science 211, 1390–1396. (10.1126/science.7466396) 7466396

[11] Packer C 1977 Reciprocal altruism in *Papio anubis*. Nature 265, 441–443. (10.1038/265441a0)

[12] Wilkinson GS 1984 Reciprocal food sharing in the vampire bat. Nature 308, 181–184. (10.1038/308181a0)

[13] Seyfarth RM, Cheney DL 1984 Grooming, alliances and reciprocal altruism in vervet monkeys. Nature 308, 541–543. (10.1038/308541a0) 6709060

[14] Fischer EA 1988 Simultaneous hermaphroditism, tit-for-tat, and the evolutionary stability of social systems. Ethol. Sociobiol. 9, 119–136. (10.1016/0162-3095(88)90017-9)

[15] Bercovitch FB 1988 Coalitions, cooperation, and reproductive tactics among adult male baboons. Anim. Behav. 36, 1198–1209. (10.1016/S0003-3472(88)80079-4)

[16] Noë R 1990 A veto game played by baboons: a challenge to the use of the Prisoner's Dilemma as a paradigm for reciprocity and cooperation. Anim. Behav. 39, 78–90. (10.1016/S0003-3472(05)80728-6)

[17] Carter GG, Wilkinson GS 2013 Does food sharing in vampire bats demonstrate reciprocity? Commun. Integr. Biol. 6, e25783 (10.4161/cib.25783) 24505498 PMC3913674

[18] Friedman JW, Hammerstein P 1991 To trade, or not to trade; that is the question. In Game equilibrium models I (ed. Selten R), pp. 257–275. Berlin, Germany: Springer.

[19] Clements KC, Stephens DW 1995 Testing models of non-kin cooperation: mutualism and the Prisoner's Dilemma. Anim. Behav. 50, 527–535. (10.1006/anbe.1995.0267)

[20] Hammerstein P 2003 Why is reciprocity so rare in social animals? A protestant appeal. In Genetic and cultural evolution of cooperation (ed. Hammerstein P), pp. 83–94. Cambridge, MA: MIT Press.

[21] Bshary R, Bronstein JL 2004 Game structures in mutualistic interactions: what can the evidence tell us about the kind of models we need? Adv. Study Behav. 34, 59–101. (10.1016/S0065-3454(04)34002-7)

[22] Sachs JL, Mueller UG, Wilcox TP, Bull JJ 2004 The evolution of cooperation. Q. Rev. Biol. 79, 135–160. (10.1086/383541) 15232949

[23] Leimar O, Hammerstein P 2006 Facing the facts. J. Evol. Biol. 19, 1403–1405. (10.1111/j.1420-9101.2006.01156.x) 16910967

[24] Bergmüller R, Johnstone RA, Russell AF, Bshary R 2007 Integrating cooperative breeding into theoretical concepts of cooperation. Behav. Process. 76, 61–72. (10.1016/j.beproc.2007.07.001) 17703898

[25] Silk J 2007 The adaptive value of sociality in mammalian groups. Phil. Trans. R. Soc. B 362, 539–559. (10.1098/rstb.2006.1994) 17363359 PMC2346516

[26] West SA, Griffin AS, Gardner A 2007 Evolutionary explanations for cooperation. Curr. Biol. 17, R661–R672. (10.1016/j.cub.2007.06.004) 17714660

[27] Clutton-Brock T 2009 Cooperation between non-kin in animal societies. Nature 462, 51–57. (10.1038/nature08366) 19890322

[28] Noë R 2006 Cooperation experiments: coordination through communication versus acting apart together. Anim. Behav. 71, 1–18. (10.1016/j.anbehav.2005.03.037)

[29] Hoeksema JD, Bruna EM 2000 Pursuing the big questions about interspecific mutualism: a review of theoretical approaches. Oecologia 125, 321–330. (10.1007/s004420000496) 28547326

[30] Wilkinson DM, Sherratt TN 2001 Horizontally acquired mutualisms, an unsolved problem in ecology? Oikos 92, 377–384. (10.1034/j.1600-0706.2001.920222.x)

[31] Bshary R, Grutter AS, Willener AST, Leimar O 2008 Pairs of cooperating cleaner fish provide better service quality than singletons. Nature 455, 964–966. (10.1038/nature07184) 18923522

[32] Krams I, Krama T, Igaune K, Mänd R 2008 Experimental evidence of reciprocal altruism in the pied flycatcher. Behav. Ecol. Sociobiol. 62, 599–605. (10.1007/s00265-007-0484-1)

[33] Brandl SJ, Bellwood DR 2015 Coordinated vigilance provides evidence for direct reciprocity in coral reef fishes. Sci. Rep. 5, 14556 (10.1038/srep14556) 26403250 PMC4585916

[34] Voelkl B, Portugal SJ, Unsöld M, Usherwood JR, Wilson AM, Fritz J 2015 Matching times of leading and following suggest cooperation through direct reciprocity during V-formation flight in ibis. Proc. Natl Acad. Sci. USA 112, 2115–2120. (10.1073/pnas.1413589112) 25646487 PMC4343164

[35] Hoeksema JD, Schwartz MW 2003 Expanding comparative-advantage biological market models: contingency of mutualism on partners’ resource requirements and acquisition trade-offs. Proc. R. Soc. Lond. B 270, 913–919. (10.1098/rspb.2002.2312) PMC1691320 12803905

[36] Johnson NC et al. 2006 From Lilliput to Brobdingnag: extending models of mycorrhizal function across scales. BioScience 56, 889–900. (10.1641/0006-3568(2006)56%5B889:FLTBEM%5D2.0.CO;2)

[37] Kummel M, Salant SW 2006 The economics of mutualisms: optimal utilization of mycorrhizal mutualistic partners by plants. Ecology 87, 892–902. (10.1890/0012-9658(2006)87%5B892:TEOMOU%5D2.0.CO;2) 16676533

[38] de Mazancourt C, Schwartz MW 2010 A resource ratio theory of cooperation. Ecol. Lett. 13, 349–359. (10.1111/j.1461-0248.2009.01431.x) 20455920

[39] Schwartz MW, Hoeksema JD 1998 Specialization and resource trade: biological markets as a model of mutualisms. Ecology 79, 1029–1038. (10.1890/0012-9658(1998)079%5B1029:SARTBM%5D2.0.CO;2)

[40] Tasoff J, Mee MT, Wang HH 2015 An economic framework of microbial trade. PLoS ONE 10, e0132907 (10.1371/journal.pone.0132907) 26222307 PMC4519184

[41] Wyatt GAK, Kiers ET, Gardner A, West SA 2014 A biological market analysis of the plant–mycorrhizal symbiosis. Evolution 68, 2603–2618. (10.1111/evo.12466) 24909843

[42] Bull JJ, Rice WR 1991 Distinguishing mechanisms for the evolution of co-operation. J. Theor. Biol. 149, 63–74. (10.1016/S0022-5193(05)80072-4) 1881147

[43] Denison RF 2000 Legume sanctions and the evolution of symbiotic cooperation by rhizobia. Am. Nat. 156, 567–576. (10.1086/316994) 29592542

[44] West SA, Kiers ET, Simms EL, Denison RF 2002 Sanctions and mutualism stability: why do rhizobia fix nitrogen? Proc. R. Soc. Lond. B 269, 685–694. (10.1098/rspb.2001.1878) PMC1690951 11934359

[45] Kiers ET, Rousseau RA, West SA, Denison RF 2003 Host sanctions and the legume–rhizobium mutualism. Nature 425, 78–81. (10.1038/nature01931) 12955144

[46] Kiers ET, Denison RF, Kawakita A, Herre EA 2011 The biological reality of host sanctions and partner fidelity. Proc. Natl Acad. Sci. USA 108, E7 (10.1073/pnas.1014546108) 21220350 PMC3024659

[47] Fleming TH, Holland JN 1998 The evolution of obligate pollination mutualisms: *Senita cactus* and *Senita moth*. Oecologia 114, 368–375. (10.1007/s004420050459) 28307780

[48] Dufaÿ M, Anstett M-C 2003 Conflicts between plants and pollinators that reproduce within inflorescences: evolutionary variations on a theme. Oikos 100, 3–14. (10.1034/j.1600-0706.2003.12053.x)

[49] Segraves KA, Althoff DM, Pellmyr O 2005 Limiting cheaters in mutualism: evidence from hybridization between mutualist and cheater yucca moths. Proc. R. Soc. B 272, 2195–2201. (10.1098/rspb.2005.3201) PMC1559949 16191630

[50] James CD, Hoffman MT, Lightfoot DC, Forbes GS, Whitford WG 1994 Fruit abortion in *Yucca elata* and its implications for the mutualistic association with yucca moths. Oikos 69, 207–216. (10.2307/3546140)

[51] Pellmyr O, Huth CJ 1994 Evolutionary stability of mutualism between yuccas and yucca moths. Nature 372, 257–260. (10.1038/372257a0)

[52] Ferdy J-B, Després L, Godelle B 2002 Evolution of mutualism between globeflowers and their pollinating flies. J. Theor. Biol. 217, 219–234. (10.1006/jtbi.2002.3018) 12202115

[53] Meyer KM, Soldaat LL, Auge H, Thulke H-H 2014 Adaptive and selective seed abortion reveals complex conditional decision making in plants. Am. Nat. 183, 376–383. (10.1086/675063) 24561600

[54] Roberts G 1998 Competitive altruism: from reciprocity to the handicap principle. Proc. R. Soc. Lond. B 265, 427–431. (10.1098/rspb.1998.0312)

[55] Barclay P 2004 Trustworthiness and competitive altruism can also solve the tragedy of the commons. Evol. Hum. Behav. 25, 209–220. (10.1016/j.evolhumbehav.2004.04.002)

[56] Hardy CL, Van Vugt M 2006 Nice guys finish first: the competitive altruism hypothesis. Pers. Soc. Psychol. Bull. 32, 1402–1413. (10.1177/0146167206291006) 16963610

[57] Barclay P, Willer R 2007 Partner choice creates competitive altruism in humans. Proc. R. Soc. B 274, 749–753. (10.1098/rspb.2006.0209) PMC2197220 17255001

[58] Kiyonari T, Barclay P 2008 Cooperation in social dilemmas: free riding may be thwarted by second-order reward rather than by punishment. J. Pers. Soc. Psychol. 95, 826–842. (10.1037/a0011381) 18808262

[59] Chiang Y-S 2010 Self-interested partner selection can lead to the emergence of fairness. Evol. Hum. Behav. 31, 265–270. (10.1016/j.evolhumbehav.2010.03.003)

[60] Macfarlan SJ 2010 The logic of labor exchange in a Dominican village: competitive altruism, biologic markets, and the nexus of male social relations. Vancouver, Canada: Washington State University.

[61] Sylwester K, Roberts G 2010 Cooperators benefit through reputation-based partner choice in economic games. Biol. Lett. 6, 659–662. (10.1098/rsbl.2010.0209) 20410026 PMC2936156

[62] Van Vugt M, Hardy CL 2010 Cooperation for reputation: wasteful contributions as costly signals in public goods. Group Process. Intergroup Relations 13, 101–111. (10.1177/1368430209342258)

[63] Barclay P 2012 Harnessing the power of reputation: strengths and limits for promoting cooperative behaviors. Evol. Psychol. 10, 868–883. (10.1177/147470491201000509) 23253792 PMC10429114

[64] Böhm R, Regner T 2012 Charitable giving among females and males: an empirical test of the competitive altruism hypothesis. In Jena economic research papers, number 2012-038. Jena, Germany: Friedrich Schiller University.

[65] Fraser B 2012 Explaining strong reciprocity: cooperation, competition, and partner choice. Biol. Theory 6, 113–119. (10.1007/s13752-012-0016-8)

[66] Sylwester K, Roberts G 2013 Reputation-based partner choice is an effective alternative to indirect reciprocity in solving social dilemmas. Evol. Hum. Behav. 34, 201–206. (10.1016/j.evolhumbehav.2012.11.009)

[67] Nolin DA 2012 Food-sharing networks in Lamalera, Indonesia: status, sharing, and signaling. Evol. Hum. Behav. 33, 334–345. (10.1016/j.evolhumbehav.2011.11.003) 22822299 PMC3398706

[68] André J-B, Baumard N 2011 The evolution of fairness in a biological market. Evolution 65, 1447–1456. (10.1111/j.1558-5646.2011.01232.x) 21521194

[69] Baumard N, Sheskin M 2015 Partner choice and the evolution of a contractualist morality. In The moral brain: a multidisciplinary perspective (eds Decety J, Wheatley T), pp. 35–48. Cambridge, MA: MIT Press.

[70] Debove S, André J-B, Baumard N 2015 Partner choice creates fairness in humans. Proc. R. Soc. Lond. B 282, 20150392 (10.1098/rspb.2015.0392) PMC4455807 25972467

[71] Barclay P 2013 Strategies for cooperation in biological markets, especially for humans. Evol. Hum. Behav. 34, 164–175. (10.1016/j.evolhumbehav.2013.02.002)

[72] Barclay P 2015 Biological markets and the effects of partner choice on cooperation and friendship. Curr. Opin. Psychol. 7, 33–38. (10.1016/j.copsyc.2015.07.012)

[73] Xu Z, Le Y, Zhang L 2014 Evolutionary prisoner's dilemma on evolving random networks. Phys. Rev. E 89, 042142 (10.1103/PhysRevE.89.042142) 24827227

[74] Ghang W, Nowak MA 2015 Indirect reciprocity with optional interactions. J. Theor. Biol. 365, 1–11. (10.1016/j.jtbi.2014.09.036) 25305556

[75] Ashlock D, Smucker MD, Stanley EA, Tesfatsion L 1996 Preferential partner selection in an evolutionary study of Prisoner's Dilemma. Biosystems 37, 99–125. (10.1016/0303-2647(95)01548-5) 8924642

[76] Stanley EA, Ashlock D, Smucker M 1995 Iterated prisoner's dilemma with choice and refusal of partners: evolutionary results. In Advances in artificial life (eds Morán F, Moreno A, Merelo J, Chacón P), pp. 490–502. Berlin, Germany: Springer.

[77] Batali J, Kitcher P 1995 Evolution of altruism in optional and compulsory games. J. Theor. Biol. 175, 161–171. (10.1006/jtbi.1995.0128) 7564396

[78] Noë R In press. How do biological markets compare to the markets of economics? In Markets and Marketization (eds Mäki U, Mäkalä P, Walsh A). Oxford, UK: Oxford University Press.

[79] Ricardo D 1817 On the principles of political economy and taxation. London, UK: John Murray.

[80] Smith SE, Read DJ 2008 Mycorrhizal symbiosis, 3rd edn Amsterdam, The Netherlands: Academic Press—Elsevier.

[81] Werner GDA et al. 2014 Evolution of microbial markets. Proc. Natl Acad. Sci. USA 111, 1237–1244. (10.1073/pnas.1315980111) 24474743 PMC3910570

[82] Janzen DH 1966 Coevolution of mutualism between ants and acacias in Central America. Evolution 20, 249–275. (10.2307/2406628) 28562970

[83] Heil M, González-Teuber M, Clement LW, Kautz S, Verhaagh M, Bueno JCS 2009 Divergent investment strategies of *Acacia* myrmecophytes and the coexistence of mutualists and exploiters. Proc. Natl Acad. Sci. USA 106, 18 091–18 096. (10.1073/pnas.0904304106) 19717429 PMC2775331

[84] Ruby EG, McFall-Ngai MJ 1999 Oxygen-utilizing reactions and symbiotic colonization of the squid light organ by *Vibrio fischeri*. Trends Microbiol. 7, 414–420. (10.1016/s0966-842x(99)01588-7) 10498950

[85] McFall-Ngai M 2008 Hawaiian bobtail squid. Curr. Biol. 18, R1043–R1044. (10.1016/j.cub.2008.08.059) 19036327

[86] McFall-Ngai M 2008 Host–microbe symbiosis: the squid-vibrio association—a naturally occurring, experimental model of animal/bacterial partnerships. Adv. Exp. Med. Biol. 635, 102–112. (10.1007/978-0-387-09550-9_9) 18841707

[87] Brusca RC, Gilligan MR 1983 Tongue replacement in a marine fish (*Lutjanus guttatus*) by a parasitic isopod (*Crustacea: Isopoda*). Copeia 3, 813–816. (10.2307/1444352)

[88] Greene E, Lyon BE, Muehter VR, Ratcliffe L, Oliver SJ, Boag PT 2000 Disruptive sexual selection for plumage coloration in a passerine bird. Nature 407, 1000–1003. (10.1038/35039500) 11069178

[89] Albo MJ, Costa FG 2010 Nuptial gift-giving behaviour and male mating effort in the Neotropical spider *Paratrechalea ornata* (Trechaleidae). Anim. Behav. 79, 1031–1036. (10.1016/j.anbehav.2010.01.018)

[90] Kaburu SSK, Newton-Fisher NE 2015 Egalitarian despots: hierarchy steepness, reciprocity and the grooming-trade model in wild chimpanzees, *Pan troglodytes*. Anim. Behav. 99, 61–71. (10.1016/j.anbehav.2014.10.018) PMC4287234 25580017

[91] Gumert MD 2007 Payment for sex in a macaque mating market. Anim. Behav. 74, 1655–1667. (10.1016/j.anbehav.2007.03.009)

[92] Hammerstein P, Leimar O 2015 Evolutionary game theory in biology. In Handbook of game theory with economic applications (eds Young HP, Shmuel Z), pp. 575–617. Amsterdam, The Netherlands: Elsevier.

[93] Parker GA, Hammerstein P 1985 Game theory and animal behaviour. In Evolution. Essays in honour of John Maynard Smith (eds Greenwood PJ, Harvey PH, Slatkin M), pp. 73–94. Cambridge, UK: Cambridge University Press.

[94] Bowles S, Hammerstein P 2003 Does market theory apply to biology? In Genetic and cultural evolution of cooperation (ed. Hammerstein P), pp. 153–165. Cambridge, MA: MIT Press.

[95] Leimar O, Hammerstein P 2010 Cooperation for direct fitness benefits. Phil. Trans. R. Soc. B 365, 2619–2626. (10.1098/rstb.2010.0116) 20679106 PMC2936167

[96] McFall-Ngai M 2014 Divining the essence of symbiosis: insights from the squid-vibrio model. PLoS Biol. 12, e1001783 (10.1371/journal.pbio.1001783) 24504482 PMC3913551

[97] Nyholm SV, McFall-Ngai MJ 2004 The winnowing: establishing the squid–*Vibrio* symbiosis. Nat. Rev. Microbiol. 2, 632–642. (10.1038/nrmicro957) 15263898

[98] Rader BA, Nyholm SV 2012 Host/microbe interactions revealed through ‘omics’ in the symbiosis between the Hawaiian bobtail squid *Euprymna scolopes* and the bioluminescent bacterium *Vibrio fischeri*. Biol. Bull. 223, 103–111. 22983036 10.1086/BBLv223n1p103

[99] Heil M, Rattke J, Boland W 2005 Postsecretory hydrolysis of nectar sucrose and specialization in ant/plant mutualism. Science 308, 560–563. (10.1126/science.1107536) 15845855

[100] Kautz S, Lumbsch HT, Ward PS, Heil M 2009 How to prevent cheating: a digestive specialization ties mutualistic plant-ants to their ant–plant partners. Evolution 63, 839–853. (10.1111/j.1558-5646.2008.00594.x) 19210534

[101] Kiers ET et al. 2011 Reciprocal rewards stabilize cooperation in the mycorrhizal symbiosis. Science 333, 880–882. (10.1126/science.1208473) 21836016

[102] Archetti M, Scheuring I, Hoffman M, Frederickson ME, Pierce NE, Yu DW 2011 Economic game theory for mutualism and cooperation. Ecol. Lett. 14, 1300–1312. (10.1111/j.1461-0248.2011.01697.x) 22011186

[103] Kiers ET, Denison RF 2008 Sanctions, cooperation, and the stability of plant–rhizosphere mutualisms. Annu. Rev. Ecol. Evol. Syst. 39, 215–236. (10.1146/annurev.ecolsys.39.110707.173423)

[104] Simms EL, Taylor DL, Povich J, Shefferson RP, Sachs JL, Urbina M, Tausczik Y 2006 An empirical test of partner choice mechanisms in a wild legume–rhizobium interaction. Proc. R. Soc. B 273, 77–81. (10.1098/rspb.2005.3292) PMC1560009 16519238

[105] Archetti M, Úbeda F, Fudenberg D, Green J, Pierce NE, Yu DW 2011 Let the right one in: a microeconomic approach to partner choice in mutualisms. Am. Nat. 177, 75–85. (10.1086/657622) 21091210

[106] Von Frisch K 1967 The dance language and orientation of bees. Cambridge, MA: Harvard University Press.

[107] Cournot A-A 1838 Recherches sur les principes mathématiques de la théorie des richesses. Paris, France: L. Hachette.

[108] Nash JF 1950 Equilibrium points in *n*-person games. Proc. Natl Acad. Sci. USA 36, 48–49. (10.1073/pnas.36.1.48) 16588946 PMC1063129

[109] Nash JF 1951 Non-cooperative games. Ann. Math. 54, 286–295. (10.2307/1969529)

[110] Maynard Smith J, Price GR 1973 The logic of animal conflict. Nature 246, 15–18. (10.1038/246015a0)

[111] Bücking H, Shachar-Hill Y 2005 Phosphate uptake, transport and transfer by the arbuscular mycorrhizal fungus *Glomus intraradices* is stimulated by increased carbohydrate availability. New Phytol. 165, 899–912. (10.1111/j.1469-8137.2004.01274.x) 15720701

[112] Bever JD, Richardson SC, Lawrence BM, Holmes J, Watson M 2009 Preferential allocation to beneficial symbiont with spatial structure maintains mycorrhizal mutualism. Ecol. Lett. 12, 13–21. (10.1111/j.1461-0248.2008.01254.x) 19019195

[113] Giovannetti M, Sbrana C, Avio L, Strani P 2004 Patterns of below-ground plant interconnections established by means of arbuscular mycorrhizal networks. New Phytol. 164, 175–181. (10.1111/j.1469-8137.2004.01145.x) 33873483

[114] Montesinos-Navarro A, Segarra-Moragues JG, Valiente-Banuet A, Verdú M 2012 The network structure of plant–arbuscular mycorrhizal fungi. New Phytol. 194, 536–547. (10.1111/j.1469-8137.2011.04045.x) 22269207

[115] Bowles S 2004 Microeconomics. Behavior, institutions and evolution. Princeton, NJ: Princeton University Press.

[116] Walras L 1874 Eléments d’économie politique pure ou théorie de la richesse sociale. Lausanne, Switzerland: L. Corbaz.

[117] Chapman T, Liddle LF, Kalb JM, Wolfner MF, Partridge L 1995 Cost of mating in *Drosophila melanogaster* females is mediated by male accessory gland products. Nature 373, 241–244. (10.1038/373241a0) 7816137

[118] Düsing K 1883 Die Factoren, welche die Sexualität entscheiden. Jena, Germany: G. Fischer.

[119] Fisher RA 1930 The genetical theory of natural selection. Oxford, UK: Clarendon Press.

[120] West S 2009 Sex allocation. Princeton, NJ: Princeton University Press.

[121] Kokko H, Jennions MD 2008 Parental investment, sexual selection and sex ratios. J. Evol. Biol. 21, 919–948. (10.1111/j.1420-9101.2008.01540.x) 18462318

[122] Bshary R 2001 The cleaner fish market. In Economics in nature. Social Dilemmas, mate choice and biological markets (eds Noë R, van Hooff JARAM, Hammerstein P), pp. 146–172. Cambridge, UK: Cambridge University Press.

[123] Fruteau C, Voelkl B, van Damme E, Noë R 2009 Supply and demand determine the market value of food providers in wild vervet monkeys. Proc. Natl Acad. Sci. USA 106, 12 007–12 012. (10.1073/pnas.0812280106) 19581578 PMC2706267

[124] Riechert SE, Hammerstein P 1983 Game theory in the ecological context. Annu. Rev. Ecol. Syst. 14, 377–409. (10.1146/annurev.es.14.110183.002113)

[125] Zahavi A 1975 Mate selection—a selection for a handicap. J. Theor. Biol. 53, 205–214. (10.1016/0022-5193(75)90111-3) 1195756

[126] Grafen A 1990 Biological signals as handicaps. J. Theor. Biol. 144, 517–546. (10.1016/S0022-5193(05)80088-8) 2402153

[127] Spence AM 1973 Job market signaling. Q. J. Econ. 87, 355–374. (10.2307/1882010)

[128] Proulx SR, Day T, Rowe L 2002 Older males signal more reliably. Proc. R. Soc. Lond. B 269, 2291–2299. (10.1098/rspb.2002.2129) PMC1691170 12495495

[129] Fraser B 2013 False advertising in biological markets: partner choice and the problem of reliability. In Evolution, cooperation and complexity (eds Sterelny K, Joyce R, Calcott B, Fraser B), pp. 153–173. Cambridge, MA: MIT Press.

[130] de Waal FBM 2000 Attitudinal reciprocity in food sharing among brown capuchin monkeys. Anim. Behav. 60, 253–261. (10.1006/anbe.2000.1471) 10973728

[131] Schino G, Aureli F 2009 Reciprocal altruism in primates: partner choice, cognition, and emotions. Adv. Study Behav. 39, 45–69. (10.1016/S0065-3454(09)39002-6)

[132] Barrett L, Henzi SP, Weingrill T, Hill RA 1999 Market forces predict grooming reciprocity in female baboons. Proc. R. Soc. Lond. B 266, 665–670. (10.1098/rspb.1999.0687)

[133] Clarke PMR, Halliday JEB, Barrett L, Henzi SP 2010 Chacma baboon mating markets: competitor suppression mediates the potential for intersexual exchange. Behav. Ecol. 21, 1211–1220. (10.1093/beheco/arq125)

[134] Kaburu SK, Newton-Fisher N 2015 Trading or coercion? Variation in male mating strategies between two communities of East African chimpanzees. Behav. Ecol. Sociobiol. 69, 1039–1052. (10.1007/s00265-015-1917-x) 26279605 PMC4535727

[135] Blanco C, Grant J, Petry NM, Simpson HB, Alegria A, Liu S-M, Hasin D 2008 Prevalence and correlates of shoplifting in the United States: results from the National Epidemiologic Survey on Alcohol and Related Conditions (NESARC). Am. J. Psychiatry 165, 905–913. (10.1176/appi.ajp.2008.07101660) 18381900 PMC4104590

[136] Queller DC, Strassmann JE 2009 Beyond society: the evolution of organismality. Phil. Trans. R. Soc. B 364, 3143–3155. (10.1098/rstb.2009.0095) 19805423 PMC2781869

